# Resolved HBV infection is an independent risk factor for enhanced systemic inflammatory responses in COVID-19 patients

**DOI:** 10.3389/fmed.2026.1835022

**Published:** 2026-07-28

**Authors:** Yifan Xu, Jia Lu, Wei Chen, Xiaoxiang Yan, Weiguo Hu, Xiaogang Xiang

**Affiliations:** 1 Department of Infectious Diseases, Ruijin Hospital, Shanghai Jiao Tong University School of Medicine, Shanghai, China; 2 Translational Laboratory of Liver Diseases, Department of Infectious Diseases, Ruijin Hospital, Shanghai Jiao Tong University School of Medicine, Shanghai, China; 3 Department of Pulmonary and Critical Care Medicine, Ruijin Hospital, Shanghai Jiao Tong University School of Medicine, Shanghai, China; 4 Department of Cardiology, Ruijin Hospital, Shanghai Jiao Tong University School of Medicine, Shanghai, China; 5 Medical Center on Aging of Ruijin Hospital, Department of Geriatrics, Ruijin Hospital, Shanghai Jiao Tong University School of Medicine, Shanghai, China

**Keywords:** COVID-19, cytokine, immune dysregulation, inflammatory response, resolved HBV infection

## Abstract

**Purpose:**

To investigate the systemic inflammatory responses and outcomes in coronavirus disease 2019 (COVID-19) patients with resolved hepatitis B virus (HBV) infection or non-HBV infection.

**Methods:**

This single-center, retrospective study was conducted in Tongji Hospital of Wuhan, China, from 20 January 2020 to 30 March 2020. A total of 215 COVID-19 cases with available test results were included. Demographic and clinical characteristics, and results of laboratory test results, were collected and analyzed.

**Results:**

Of the 215 included patients, the numbers of patients with current HBV infection, resolved HBV infection, and non-HBV infection were 15, 134, and 66, respectively. Inflammatory biomarkers, such as C-reactive protein (CRP), procalcitonin (PCT), and ferritin, were significantly elevated in the resolved HBV infection group, as well pro-inflammatory cytokines, including interleukin-6 (IL-6), soluble interleukin-2 receptor (sIL-2R), tumor necrosis factor alpha (TNF-*α*), and IL-8. Resolved HBV infection was independently associated with elevated proinflammatory cytokines. Patients with resolved HBV infection (59.7%) were more likely to develop into a severe state compared to patients with current HBV infection (46.7%) and non-HBV infection (43.9%).

**Conclusion:**

Resolved HBV infection is an independent risk factor for patients with COVID-19. COVID-19 patients with resolved HBV infection are more likely to have enhanced systemic inflammatory responses and worse prognoses than those with non-HBV infection.

## Introduction

Coronavirus disease 2019 (COVID-19) is caused by severe acute respiratory syndrome coronavirus 2 (SARS-CoV-2), which is a third documented highly pathogenic coronavirus (1). Globally, there have been 620,878,405 confirmed cases of COVID-19, including 6,543,138 deaths till 14 October 2022 (2). Most patients with COVID-19 have mild and moderate symptoms, but severe cases can deteriorate to acute respiratory distress syndrome (ARDS) and multiple organ dysfunction syndrome.

It is estimated that two billion people alive worldwide have ever been infected by the hepatitis B virus (HBV) in their lives (3). HBV is mainly transmitted through perinatal exposure, percutaneous or mucosal contact with infectious blood or body fluids, and sexual contact. Common risk factors for HBV infection include infants born to an HBV- infected mother, unprotected sexual activity, injection-drug use or needle sharing, unsafe injections or blood transfusions, occupational exposure to blood, and close household contact with HBV-infected individuals (4). These exposure patterns may result in either current infection or evidence of previous exposure.

HBV infection status is primarily assessed using serological biomarkers and HBV DNA testing. Hepatitis B surface antigen (HBsAg) indicates current HBV infection, whereas hepatitis B surface antibody (anti-HBs) reflects immunity acquired through vaccination or recovery. Hepatitis B core antibody (anti-HBc) suggests previous or ongoing exposure to HBV. In addition, hepatitis B e antigen (HBeAg) is commonly associated with active viral replication and high infectivity. In contrast, antibody to hepatitis B e antigen (anti-HBe) generally indicates reduced viral replication and lower infectivity. Current screening recommendations support the use of a triple-panel test including HBsAg, anti-HBs, and total anti-HBc for the initial assessment of HBV infection in adults (4). Moreover, HBV DNA quantification serves as a direct marker of viral replication and is useful for evaluating infectivity, treatment eligibility, disease progression, and reactivation risk (5).

HBV infection resolves in over 90% of immunocompetent adults (3), and individuals with resolved HBV infection are usually seropositive for anti-hepatitis B core antigen (anti-HBc) (6). Resolved HBV infection is defined by the presence of anti-HBc with/without anti-hepatitis B surface antigen (anti-HBs), negative hepatitis B surface antigen (HBsAg), undetectable serum HBV DNA, and normal levels of alanine aminotransferase (ALT) (7). Several studies have reported that the prevalence of anti-HBc ranges from 9.08–34.1% in China (8–10) and 4.6% in America (11). Spontaneous resolution of HBV infection is associated with immune control and inflammatory response against the virus (3). However, evidence regarding HBV and COVID-19 co-infection remains inconsistent. Recent studies have suggested that HBV infection may be associated with severe COVID-19 outcomes, liver injury, or increased mortality, although findings remain controversial. Most previous studies have focused on chronic or overall HBV infection, whereas evidence on resolved HBV infection remains limited (12–15). As severe deterioration in some COVID-19 patients is closely related to the release of excessive cytokines caused by dysregulated immune response, whether resolved HBV infection affects immune response and survival in COVID-19 patients remains to be elucidated. To evaluate whether resolved HBV infection is a risk factor for COVID-19, we conducted a retrospective study to analyze the clinical characteristics, biochemical parameters, and inflammatory cytokine levels in COVID-19 patients with resolved HBV infection or non-HBV infection.

## Patients and methods

### Study design and participants

This single-center retrospective study included hospitalized patients with COVID-19 treated at Tongji Hospital, Tongji Medical College, Huazhong University of Science and Technology, Wuhan, China, between 20 January 2020 and 30 March 2020. During the COVID-19 outbreak in Wuhan, the Ruijin Hospital Aid Hubei Province COVID-19 Critical Care Medical Team was dispatched to Tongji Hospital on 9 February 2020, as part of the national emergency medical response coordinated by the National Health Commission of China. Some patients later managed by this team had been admitted before its arrival, with hospitalization dates as early as January 20, 2020. The team was organized by Ruijin Hospital and consisted of physicians from infectious diseases, critical care, respiratory medicine, and other relevant specialties, as well as nurses and administrative/logistical staff. In addition, part of the ventilators and medications used in clinical care were transported from Ruijin Hospital to Wuhan to support frontline treatment. The study investigators retrospectively extracted clinical data from the electronic medical records of eligible patients with available HBV serological results. In view of the available serological results of HBV tests, two hundred and fifteen patients with COVID-19 from 20 January 2020 to 30 March 2020 in Tongji Hospital, Tongji Medical College, Huazhong University of Science and Technology were finally included in the study. The diagnosis and disease severity of COVID-19 were based on the New Coronavirus Pneumonia Prevention and Control Program (Trial Version 5) published by the National Health Commission of China (16). Patients with COVID-19 were diagnosed with positive results on a reverse-transcription polymerase chain reaction (RT-PCR) assay of a throat swab for testing SARS-CoV-2. The included inpatients were diagnosed with COVID-19 concurrent with chronic HBV infection, resolved HBV infection, or non-HBV infection, according to the World Health Organization interim guidance and the guidelines of the American Association for the Study of Liver Diseases (5, 17). This study was conducted within the framework of the national emergency medical response to the COVID-19 outbreak in Wuhan, with authorization from the National Health Commission of China, Tongji Hospital, and Ruijin Hospital. As patient care at the study site was delivered by the Ruijin Hospital Aid Hubei Province COVID-19 Critical Care Medical Team, the study was reviewed and approved by the Institutional Review Board of Ruijin Hospital, Shanghai Jiao Tong University School of Medicine (ETHICS No: [2020] Linlun No. 34th).

### Data collection

Demographic characteristics, COVID-19 symptoms at admission, medications administered, laboratory test results, and treatment outcomes were extracted from electronic medical records. HBV test results were collected at admission. Resolved HBV-infected individuals were defined as follows: (1) seronegative HBsAg; (2) seropositive anti-HBc with or without positive anti-HBs. Seropositive HBsAg, and none-HBV infected individuals diagnosed current HBV-infected individuals were diagnosed by seronegative HBsAg with negative anti-HBc (18). Liver injury was defined as ALT and/or AST > 3 × upper limits of normal (ULN) and/or TBIL > 2 × ULN (19).

### Statistical analysis

Continuous variables were presented as median and interquartile range (IQR), and categorical variables as counts and percentages (%). For comparisons across the three groups, the Kruskal–Wallis test was used for continuous variables. In contrast, the Pearson’s chi-square test or Fisher’s exact test was used for categorical variables based on the data characteristics. If the overall intergroup difference of continuous variables reached statistical significance, *post hoc* pairwise comparisons were subsequently conducted using Dunn’s test with Bonferroni correction. A *p*-value < 0.05 was defined as statistically significant. To identify factors associated with elevated cytokine levels, univariable logistic regression analysis was first conducted. Variables with *p* < 0.1 in the univariable analysis were subsequently entered into the multivariable logistic regression model. Statistical analyses were performed using SPSS Statistics 25 (IBM, Armonk, NY, USA) and GraphPad Prism version 9.5.0 (GraphPad Software, San Diego, CA, USA).

## Results

### Demographic and clinical characteristics of the patients

Among the 215 cases of COVID-19 patients with detailed results of HBV tests, 15 cases were diagnosed as current HBV infection, 134 cases were diagnosed as resolved HBV infection, and 66 were non-HBV infection (Supplementary Figure S1). Patients with resolved HBV infection (65.0, 57.0–72.0) were older than current HBV-infected patients (61.0, 55.5–68.0) and non-HBV-infected patients (58.5, 47.0–68.0). Most patients had fever (167, 77.7%) and cough (170, 79.1%) at admission. Hypertension (84, 39.1%) and diabetes (40, 18.6%) were the two common comorbidities among all the patients. Only 1 patient (1 case, 0.5%) with current HBV infection was taking the oral anti-HBV drug Entecavir. Antiviral drugs, including Arbidol (169 cases, 78.6%), Oseltamivirphosphate (48 cases, 22.3%), Lopinavir (21 cases, 9.8%), Ritonavir (21 cases, 9.8%), and Ganciclovir (3 cases, 1.4%), were used in patients. Cephalosporins (49 cases, 22.8%), Moxifloxacin (59 cases, 27.4%), and Levofloxacin (41 cases, 19.1%) were used for anti-bacterial treatment in some patients. Importantly, patients with resolved HBV infection (31 cases, 23.1%) who received glucocorticoids were more common than patients with current HBV infection (1 case, 6.7%) or non-HBV infection (7 cases, 10.6%) (Table 1).

**Table 1:** Demographic characteristics of the included COVID-19 patients.

Characteristics	All patients (*N* = 215)	Current HBV infection (*N* = 15)	Resolved HBV infection (*N* = 134)	Non-HBV infection (*N* = 66)	H-value/χ^2^	*p*-value
Age (median, IQR)	64.0 (55.0, 71.0)	61.0 (55.5, 68.0)	65.0 (57.0, 72.0)	58.5 (47.0, 68.0)	8.7628	**0.0125**
Gender						
Female, *n* (%)	121 (56.3%)	5 (33.3%)	81 (60.4%)	35 (53.0%)	4.4391	0.1087
Male, *n* (%)	94 (43.7%)	10 (66.7%)	53 (39.6%)	31 (47.0%)		
Symptoms of COVID-19						
Fever, *n* (%)	167 (77.7%)	13 (86.7%)	103 (76.9%)	51 (77.3%)	0.7561	0.6852
Cough, *n* (%)	170 (79.1%)	13 (86.7%)	106 (79.1%)	51 (77.3%)	0.652	0.7218
Expectoration, *n* (%)	107 (49.8%)	7(46.7%)	73 (54.5%)	27 (40.9%)	3.3185	0.1903
Throat pain, *n* (%)	35 (16.3%)	0 (0.0%)	23 (17.2%)	12 (18.2%)	3.169	0.205
Headache, *n* (%)	23 (10.7%)	0 (0.0%)	19 (14.2%)	4 (6.1%)	4.9825	0.0828
Dizzy, *n* (%)	21 (9.8%)	3 (20.0%)	11 (8.2%)	7 (10.6%)	2.204	0.3322
Muscular soreness, *n* (%)	40 (18.6%)	3 (20.0%)	25 (18.7%)	12 (18.2%)	0.0273	0.9864
Feeble, *n* (%)	66 (30.7%)	5 (33.3%)	38 (28.4%)	23 (34.8%)	0.9282	0.6287
Chest pain, *n* (%)	42 (19.5%)	1 (6.7%)	29 (21.6%)	12 (18.2%)	2.0355	0.3614
Chest distress, *n* (%)	86 (40.0%)	4 (26.7%)	57 (42.5%)	25 (37.9%)	1.5943	0.4506
Diarrhea, *n* (%)	50 (23.3%)	2 (13.3%)	38 (28.4%)	10 (15.2%)	5.211	0.0739
Nausea and vomiting, *n* (%)	24 (11.2%)	0 (0.0%)	18 (13.4%)	6 (9.1%)	2.8668	0.2385
Comorbidities						
Diabetes, *n* (%)	40 (18.6%)	1 (6.7%)	30 (22.4%)	9 (13.6%)	3.7541	0.153
Hypertension, *n* (%)	84 (39.1%)	6 (40.0%)	58 (43.3%)	20 (30.3%)	3.1358	0.2085
Coronary heart disease, *n* (%)	25 (11.6%)	2 (13.3%)	18 (13.4%)	5 (7.6%)	1.5219	0.4672
Stroke, *n* (%)	7 (3.3%)	0 (0.0%)	7 (5.2%)	0 (0.0%)	4.3737	0.1123
Chronic obstructive pulmonary disease, *n* (%)	10 (4.7%)	0 (0.0%)	9 (6.7%)	1 (1.5%)	3.4841	0.1752
Chronic kidney disease, *n* (%)	2 (0.9%)	0 (0.0%)	2 (1.5%)	0 (0.0%)	1.2203	0.5433
Asthma, *n* (%)	3 (1.4%)	0 (0.0%)	1 (0.7%)	2 (3.0%)	1.9048	0.3858
Rheumatism, *n* (%)	3 (1.4%)	0 (0.0%)	2 (1.5%)	1 (1.5%)	0.2283	0.8921
Malignant tumor, *n* (%)	8 (3.7%)	0 (0.0%)	5 (3.7%)	3 (4.5%)	0.705	0.7029
Drugs of treatment						
Oral HBV antiviral drug						
Entecavir, *n* (%)	1 (0.5%)	1 (6.7%)	0 (0.0%)	0 (0.0%)	13.3956	**0.0012**
Antiviral drug						
Arbidol, *n* (%)	169 (78.6%)	13 (86.7%)	101 (75.4%)	55 (83.3%)	2.2893	0.3183
Oseltamivir phosphate, *n* (%)	48 (22.3%)	3 (20.0%)	28 (20.9%)	17 (25.8%)	0.6531	0.7214
Lopinavir, *n* (%)	21 (9.8%)	1 (6.7%)	11 (8.2%)	9 (13.6%)	1.6539	0.4374
Ritonavir, *n* (%)	21 (9.8%)	1 (6.7%)	11 (8.2%)	9 (13.6%)	1.6539	0.4374
Ganciclovir, *n* (%)	3 (1.4%)	1 (6.7%)	1 (0.7%)	1 (1.5%)	3.4466	0.1785
Anti-bacterial drug						
Cephalosporins, *n* (%)	49 (22.8%)	3 (20.0%)	38 (28.4%)	8 (12.1%)	6.6966	**0.0351**
Moxifloxacin, *n* (%)	59 (27.4%)	4 (26.7%)	40 (29.9%)	15 (22.7%)	1.1318	0.5678
Levofloxacin, *n* (%)	41 (19.1%)	2 (13.3%)	30 (22.4%)	9 (13.6%)	2.5384	0.2811
Glucocorticoids, *n* (%)	39 (18.1%)	1 (6.7%)	31 (23.1%)	7 (10.6%)	6.1035	**0.0473**
Gamma globulin, *n* (%)	28 (13.0%)	1 (6.7%)	22 (16.4%)	5 (7.6%)	3.6274	0.163
Lian Hua Qing Wen capsule, *n* (%)	131 (60.9%)	11 (73.3%)	72 (53.7%)	48 (72.7%)	7.745	**0.0208**
Diagnostic classification of COVID-19						
Mild and moderate, *n* (%)	99 (46.0%)	8 (53.3%)	54 (40.3%)	37 (56.1%)	4.7668	0.0922
Severe and critically ill, *n* (%)	116 (54.0%)	7 (46.7%)	80 (59.7%)	29 (43.9%)		

Demographic characteristics of the 215 COVID-19 patients with current HBV infection, resolved HBV infection, and non-HBV infection are presented above. Data are presented as median (IQR) or *n* (%). For continuous variables, the statistical value shown in the “H-value/χ^2^” column is the H value from the Kruskal-Wallis test. For categorical variables, intergroup comparisons were performed using Pearson’s chi-squared test, and the χ^2^ value is reported. *p*-values are reported to four decimal places, and *p* < 0.0001 is presented as such. Bold values indicate statistically significant *p*-values (*p* < 0.05).

### Laboratory test and inflammatory-related biomarkers

**Figure 1:**
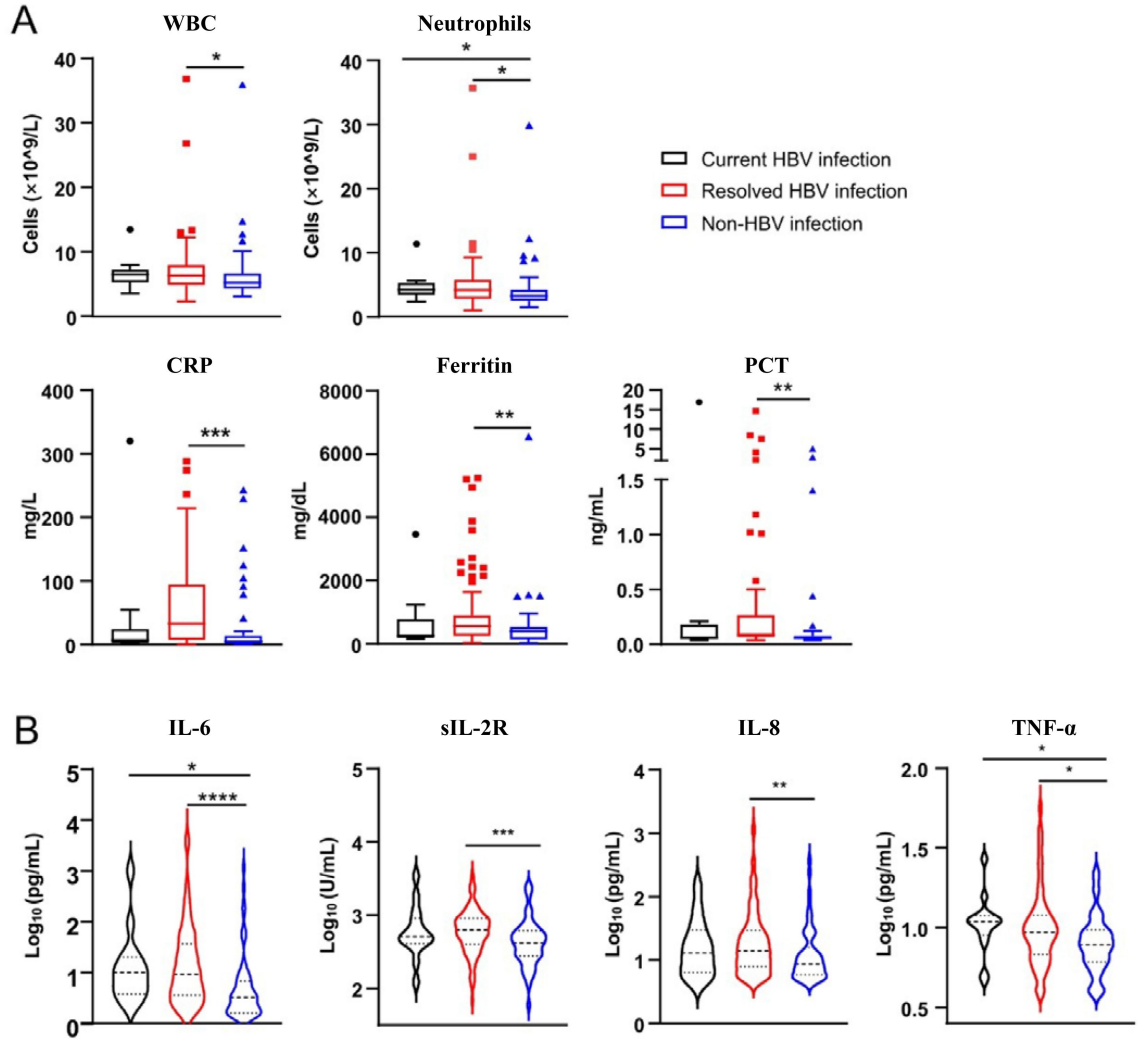
Inflammatory related biomarkers and cytokines in COVID-19 patients. **(A)** Inflammatory cells (including white blood cells and neutrophils) and inflammatory biomarkers (including CRP, ferritin, and PCT) in patients with current HBV infection, resolved HBV infection, and non-HBV infection. **(B)** Inflammatory cytokine levels (including IL-6, sIL-2R, IL-8, and TNF-*α*) in patients with current HBV infection, resolved HBV infection, and non-HBV infection. Statistical comparisons were conducted by using two-sided Kruskal-Wallis test. “*” represents *p* < 0.05; “**” represents *p* < 0.01; “***” represents *p* < 0.001; “****” represents *p* < 0.0001.

Compared with non-HBV-infected patients, elevated levels of white blood cell count (WBC, ×10^9^/L) were observed in current HBV-infected and resolved HBV-infected patients (5.25 vs. 6.47 vs. 6.18, *p* = 0.0444). Peak D-Dimer levels in current and resolved HBV-infected patients were increased substantially (*p* = 0.0001). Levels of routine inflammatory markers, including serological CRP, ferritin, and PCT, were also significantly elevated in resolved HBV-infected patients compared to non-HBV-infected patients (Figure 1). Moreover, in contrast to non-HBV-infected patients, the levels of interleukin-6 (IL-6), soluble interleukin-2 receptor (sIL-2R), IL-8, and tumor necrosis factor alpha (TNF-*α*) were also significantly upregulated in resolved HBV-infected patients (Figure 1). Compared to non-HBV-infected patients, peak AST levels were significantly elevated in the resolved HBV-infected patients (resolved HBV infection vs. non-HBV infection, 29.00 vs. 23.00, adjusted *p* = 0.013). The proportion of patients with ALT over 3 upper limit of normal (ULN) (current HBV infection vs. resolved HBV infection vs. non-HBV infection, 6.7% vs. 16.4% vs. 6.1%), AST over 3 ULN (current HBV infection vs. resolved HBV infection vs. non-HBV infection, 6.7% vs. 9.7% vs. 3.0%), and TBIL over 2 ULN (current HBV infection vs. resolved HBV infection vs. non-HBV infection, 0.0% vs. 1.5% vs. 0.0%) were higher in resolved HBV infection group. In addition, *post hoc* analyses were performed only for continuous variables with statistically significant overall differences in the initial Kruskal-Wallis test shown in Table 2 (Table 3). Specifically, a significant age difference was observed between the resolved HBV infection group and the non-HBV infection group. It was also demonstrated that significantly higher levels of neutrophil count, peak D-Dimer, peak CRP, peak PCT, peak ferritin, IL-6, sIL-2R, TNF-*α*, IL-8, and AST were observed in the resolved HBV infection group compared with the non-HBV infection group after Bonferroni correction. Furthermore, significantly higher levels of IL-6 and TNF-α were observed in the current HBV infection group than in the non-HBV infection group.

**Table 2:** Results of laboratory tests and clinical outcomes of the included COVID-19 patients.

Variables	Current HBV infection (*N* = 15)	Resolved HBV infection (*N* = 134)	Non-HBV infection (*N* = 66)	H-value/χ^2^	*p*-value
First blood routine examination (Median, IQR)					
White blood cell count (10^9/L)	6.47 (5.49, 7.17)	6.18 (4.88, 7.85)	5.25 (4.28, 6.56)	6.2275	**0.0444**
Neutrophil count (10^9/L)	4.23 (3.43, 5.01)	4.16 (2.81, 5.77)	3.41 (2.52, 4.24)	8.7773	**0.0124**
Lymphocyte count (10^9/L)	1.30 (0.98, 1.86)	1.19 (0.83, 1.65)	1.33 (1.12, 1.70)	3.3247	0.1897
Monocyte count (10^9/L)	0.60 (0.44, 0.78)	0.50 (0.37, 0.67)	0.47 (0.38, 0.61)	1.4312	0.4889
Eosinophil count (10^9/L)	0.06 (0.01, 0.13)	0.05 (0.02, 0.12)	0.08 (0.04, 0.12)	4.3270	0.1149
Basophilic granulocyte count (10^9/L)	0.02 (0.01, 0.03)	0.02 (0.01, 0.03)	0.02 (0.01, 0.04)	4.0146	0.1344
Red blood cells (10^12/L)	4.05 (3.64, 4.34)	3.92 (3.46, 4.37)	4.18 (3.68, 4.46)	4.9433	0.0844
Hemoglobin (g/L)	121.0 (110.5, 134.0)	121.5 (110.0, 134.0)	126.5 (116.0, 142.0)	5.0818	0.0788
Platelet (10^9/L)	240.0 (205.0, 352.0)	230.0 (174.0, 318.0)	253.0 (200.0, 318.0)	1.3418	0.5112
Coagulation function (Median, IQR)					
Peak INR	1.04 (1.00, 1.10)	1.06 (1.02, 1.15)	1.05 (0.99, 1.08)	5.7174	0.0573
Peak D-Dimer	1.67 (0.69, 2.21)	1.25 (0.49, 3.77)	0.59 (0.25, 0.97)	18.3302	**0.0001**
Inflammatory markers (Median, IQR)					
Peak CRP (mg/L)	7.5 (4.0, 24.6)	31.9 (5.3, 84.8)	4.6 (1.4, 14.5)	14.5679	**0.0007**
Peak PCT (ng/mL)	0.07 (0.05, 0.21)	0.09 (0.07, 0.26)	0.06 (0.05, 0.08)	13.7347	**0.0010**
Peak ferritin (mg/dL)	258.2 (191.8, 720.9)	558.0 (255.0, 915.7)	388.9 (155.1, 526.5)	8.2051	**0.0165**
Cytokine levels when IL-6 peaked (Median, IQR)					
IL-6 (pg/mL)	10.16 (4.40, 17.51)	7.76 (3.10, 30.53)	3.35 (1.58, 6.73)	17.4836	**0.0002**
sIL-2R (U/mL)	509.0 (421.0, 737.5)	618.0 (400.5, 900.5)	417.0 (280.0, 585.5)	13.7936	**0.0010**
TNF-α(pg/mL)	10.90 (9.75, 11.55)	9.50 (6.95, 11.95)	7.75 (6.20, 9.65)	10.6022	**0.0050**
IL-8 (pg/mL)	10.40 (7.10, 19.35)	14.75 (8.00, 27.35)	8.35 (5.85, 14.65)	8.8895	**0.0117**
Liver test when ALT peaked					
ALT (U/L) (Median, IQR)	29.0 (22.0, 33.0)	30.0 (19.0, 65.0)	25.0 (14.0, 40.0)	4.2022	0.1223
Normal	11 (73.3%)	72 (53.7%)	40 (60.6%)		
1–2 × ULN, *n* (%)	3 (20.0%)	30 (22.4%)	18 (27.3%)		
2–3 × ULN, *n* (%)	0 (0.0%)	10 (7.5%)	4 (6.1%)		
>3 × ULN, *n* (%)	1 (6.7%)	22 (16.4%)	4 (6.1%)		
AST (U/L) (Median, IQR)	25.0 (22.0, 36.0)	29.0 (20.0, 47.0)	23.0 (17.0, 32.0)	8.2454	**0.0162**
Normal	11 (73.3%)	79 (59.0%)	50 (75.8%)		
1–2 × ULN, *n* (%)	3 (20.0%)	32 (23.9%)	13 (19.7%)		
2–3 × ULN, *n* (%)	0 (0.0%)	10 (7.5%)	1 (1.5%)		
>3 × ULN, *n* (%)	1 (6.7%)	13 (9.7%)	2 (3.0%)		
Total bilirubin (μmol/L)	6.6 (6.1, 8.5)	7.8 (5.8, 11.8)	7.9 (6.1, 11.9)	0.8147	0.6654
Normal	14 (93.3%)	128 (95.5%)	64 (97.0%)		
1–2 × ULN, *n* (%)	1 (6.7%)	3 (2.2%)	2 (3.0%)		
>2 × ULN, *n* (%)	0 (0.0%)	2 (1.5%)	0 (0.0%)		
Direct bilirubin (μmol/L)	3.7 (2.9, 4.7)	3.5 (2.6, 5.0)	3.3 (2.6, 4.5)	0.5915	0.7440
Alkaline phosphatase (U/L)	63.0 (52.0, 81.0)	69.5 (57.0, 87.0)	61.0 (53.0, 76.0)	6.7782	**0.0337**
Treatment outcome					
Remission or recovery, *n* (%)	14 (93.3%)	126 (94.0%)	65 (98.5%)	2.1267	0.3453
Death, *n* (%)	1 (6.7%)	8 (6.0%)	1 (1.5%)		

Results of laboratory tests, inflammation levels, and clinical outcomes are presented above. Data are presented as median (IQR) or *n* (%). For continuous variables, the statistical value shown in the “H-value/χ^2^” column is the H value from the Kruskal-Wallis test. For categorical variables, intergroup comparisons were performed using Pearson’s chi-squared test, and the χ^2^ value is reported. For continuous variables with significant overall differences among the three groups, post hoc pairwise comparisons were further performed using Dunn’s test with Bonferroni correction. *p*-values are reported to four decimal places, and *p* < 0.0001 is presented as such. Bold values indicate statistically significant *p*-values (*p* < 0.05).

**Table 3:** *Post hoc* pairwise comparisons for continuous variables (Dunn-Bonferroni).

Variable	Current HBV vs. Resolved HBV	Current HBV vs. Non-HBV	Resolved HBV vs. non-HBV
Age	0.890	1.000	**0.011**
White blood cell count	1.000	0.401	**0.050**
Neutrophil count	1.000	0.259	**0.013**
Peak D-Dimer	1.000	0.099	**<0.001**
Peak CRP	0.661	1.000	**<0.001**
Peak PCT	0.750	1.000	**<0.001**
Peak ferritin	0.583	1.000	**0.017**
IL-6	1.000	**0.023**	**<0.001**
sIL-2R	1.000	0.438	**<0.001**
TNF-α	0.625	**0.022**	**0.021**
IL-8	1.000	1.000	**0.009**
AST	1.000	0.690	**0.013**
Alkaline phosphatase	0.884	1.000	**0.034**

Values are Bonferroni-adjusted *p*-values from post hoc pairwise comparisons using Dunn’s test. Post hoc analyses were performed only for continuous variables with statistically significant overall differences in the initial Kruskal–Wallis test shown in Table 2. *p*-values are reported to three decimal places, and *p* < 0.001 is presented as such. Bold values indicate statistically significant *p*-values (*p* < 0.05).

### Diagnostic classification and clinical outcomes of patients

Compared with current HBV-infected patients (7 cases, 46.7%) and non-HBV-infected patients (29 cases, 43.9%), patients with resolved HBV infection had a higher proportion of severe and critical COVID-19, reaching 59.7% (80 cases, 59.7%) (Table 1). Most patients got remission or recovery. However, 10 patients died: one (6.7%) patient in the current HBV infection group, eight (6.0%) patients in the resolved HBV infection group, and one (1.5%) patient in the non-HBV infection group (Table 2). In the 10 patients who died, 8 patients had comorbidities. Three of the eight deceased patients with resolved HBV infection had liver injury: patient 2 (ALT, 107 U/L; AST, 211 U/L); patient 8 (ALT, 738 U/L; AST, 4171 U/L; TBIL, 67.8 μmol/L); patient 9 (AST, 125 U/L; TBIL, 46.1 μmol/L). Notably, elevated cytokine levels were observed in most deceased patients, except for one patient whose cytokine levels were not measured in time (Supplementary Table S1). The deceased patient with current HBV infection also endured an inflammatory cytokine storm, and liver injury was not detected in this patient, but tuberculosis deteriorated before death (Supplementary Table S1).

### The relationship between resolved HBV infection and cytokine levels/clinical characteristics

To investigate whether elevated cytokine levels are independently associated with resolved HBV infection, univariable logistic regression analyses were first performed to screen potential candidate variables. Variables with *p* < 0.1 in the univariable analysis were subsequently entered into the multivariable logistic regression model. Factors related to cytokine levels were included. In multivariable analysis, resolved HBV infection (odds ratio [OR], 2.391; 95% confidence interval [CI], 1.027–5.567; *p* = 0.0431), age (OR, 1.051; 95% CI, 1.018–1.086; *p* = 0.0025), anti-virus drug (OR, 0.171; 95% CI, 0.050–0.580; *p* = 0.0046), and anti-bacterial drug (OR, 7.610; 95% CI, 3.358–17.248; *p* < 0.0001) were independently associated with the elevated levels of IL-6 *** (Table 4 (a)). We also found that serological sIL-2R was independently associated with age (OR, 1.074; 95% CI, 1.037–1.112; *p* < 0.0001) and anti-bacterial drug (OR, 4.975; 95% CI, 2.210–11.199; *p* = 0.0001), but not resolved HBV infection, in multivariable analysis (Table 4 (b)). TNF-*α* was also associated with resolved HBV infection (OR, 2.408; 95% CI, 1.184–4.897; *p* = 0.0152), age (OR, 1.043; 95% CI, 1.017–1.070; *p* = 0.0012) and male sex (OR, 2.524; 95% CI, 1.259–5.061; *p* = 0.0091) (Table 4 (c)), while elevated IL-8 levels were not associated with resolved HBV infection (OR, 2.079; 95% CI, 0.658–6.568; *p* = 0.2123) (Supplementary Table S2). Moreover, the relationships between relative quantitative levels of anti-HBc and levels of sIL-2R, TNF-*α*, IL-6, and IL-8 were plotted. The levels of IL-6, TNF-α, and sIL-2R were positively correlated with anti-HBc (Supplementary Figure S2).

**Table 4:** Univariable and multivariable analyses by logistic regression on factors associated with the elevated levels of IL-6, sIL-2R, and TNF-*α* in patients.

(a) Factors associated with IL-6 levels in patients				
Parameters	Univariate analysis		Multivariate analysis	
	OR (95% CI)	*p*-value	OR (95% CI)	*p*-value
Non-HBV	Ref			
Resolved HBV infection	3.833 (1.904–7.719)	**0.0002**	2.391 (1.027–5.567)	**0.0431**
Age	1.055 (1.028–1.083)	**<0.0001**	1.051 (1.018–1.086)	**0.0025**
Male sex	1.939 (1.060–3.549)	**0.0317**	1.840 (0.837–4.045)	0.1291
Diabetes	1.831 (0.888–3.775)	0.1014		
Hypertension	2.059 (1.112–3.812)	**0.0216**	1.232 (0.542–2.797)	0.6184
Coronary heart disease	1.208 (0.485–3.010)	0.6856		
Stroke	1.772 (0.385–8.161)	0.4630		
COPD	3.233 (0.808–12.942)	0.0973	2.096 (0.439–10.012)	0.3536
Anti-virus drug	0.332 (0.134–0.822)	**0.0172**	0.171 (0.050–0.580)	**0.0046**
Anti-bacterial drug	7.741 (3.880–15.440)	**<0.0001**	7.610 (3.358–17.248)	**<0.0001**
Logistic regression was performed on factors associated with elevated IL-6 levels in patients. Variables with *p* < 0.1 in the univariable analysis were entered into the multivariable model. Resolved HBV infection, age, anti-virus drug, and anti-bacterial drug were independently associated with elevated IL-6 levels. *p* < 0.05 was considered statistically significant. Bold values indicate statistically significant *p*-values (*p* < 0.05).				

(b) Factors associated with sIL-2R levels in patients				
Parameters	Univariate analysis		Multivariate analysis	
	OR (95% CI)	*p*-value	OR (95% CI)	*p-*value
Non-HBV	Ref			
Resolved HBV infection	3.286 (1.515–7.126)	**0.0026**	1.904 (0.789–4.596)	0.1521
Age	1.077 (1.043–1.112)	**<0.0001**	1.074 (1.037–1.112)	**<0.0001**
Male sex	1.303 (0.689–2.466)	0.4152		
Diabetes	1.149 (0.537–2.459)	0.7208		
Hypertension	1.670 (0.875–3.188)	0.1201		
Coronary heart disease	0.840 (0.308–2.294)	0.7338		
Stroke	2.208 (0.431–11.300)	0.3419		
COPD	3.480 (0.941–12.870)	0.0617	3.033 (0.755–12.177)	0.1178
Anti-virus drug	0.857 (0.340–2.158)	0.7436		
Anti-bacterial drug	5.313 (2.548–11.078)	**<0.0001**	4.975 (2.210–11.199)	**0.0001**
Logistic regression was performed on factors associated with elevated sIL-2R levels in patients. Variables with *p* < 0.1 in the univariable analysis were entered into the multivariable model. Age and anti-bacterial drug were independently associated with elevated sIL-2R levels. *p* < 0.05 was considered statistically significant. Bold values indicate statistically significant *p*-values (*p* < 0.05).				

(c) Factors associated with TNF-α levels in patients				
Parameters	Univariate analysis		Multivariate analysis	
	OR (95% CI)	*p-*value	OR (95% CI)	*p*-value
Non-HBV	Ref			
Resolved HBV infection	2.728 (1.435–5.186)	**0.0022**	2.408 (1.184–4.897)	**0.0152**
Age	1.046 (1.021–1.071)	**0.0003**	1.043 (1.017–1.070)	**0.0012**
Male sex	2.080 (1.119–3.866)	**0.0206**	2.524 (1.259–5.061)	**0.0091**
Diabetes	1.160 (0.557–2.414)	0.6918		
Hypertension	1.395 (0.748–2.603)	0.2950		
Coronary heart disease	1.219 (0.478–3.110)	0.6789		
Stroke	0.724 (0.142–3.695)	0.6982		
COPD	1.762 (0.440–7.056)	0.4233		
Anti-virus drug	1.058 (0.437–2.563)	0.9011		
Anti-bacterial drug	1.818 (0.991–3.334)	0.0534	1.140 (0.576–2.257)	0.7067

Logistic regression was performed on factors associated with elevated TNF-α levels in patients. Variables with *p* < 0.1 in the univariable analysis were entered into the multivariable model. Resolved HBV infection, age, and male sex were independently associated with elevated TNF-α levels. *p* < 0.05 was considered statistically significant. Bold values indicate statistically significant *p*-values (*p* < 0.05).

## Discussion

This retrospective study was the first to demonstrate that COVID-19 patients with resolved HBV infection had higher inflammatory responses than those with non-HBV infection. Our study showed that resolved HBV infection was independently associated with elevated levels of proinflammatory cytokines, including IL-6 and TNF-α, in multivariable logistic regression analyses. In contrast, elevated sIL-2R was mainly associated with age and anti-bacterial drug use. Candidate variables were initially screened using univariable analysis, and variables with *p* < 0.1 were subsequently included in the multivariable models. Moreover, glucocorticoid therapy in resolved HBV-infected patients with COVID-19 was more likely to be used in the present cohort. Liver injury and inflammatory cytokine storm-related death were more common in COVID-19 patients with resolved HBV infection.

The impact of HBV infection on patients with COVID-19 remains controversial (18). Some studies have reported that patients with SARS-CoV-2 and HBV co-infection are at increased risk of liver injury, and inflammatory abnormalities, including elevated IL-6 and D-dimer, and lactate dehydrogenase levels, might contribute to this process (20). However, findings from large cohort studies remain inconsistent. A territory-wide cohort from Hong Kong reported that neither current nor past HBV infection was associated with increased mortality or acute liver injury in patients with COVID-19 (18). Similarly, a Korean nationwide cohort did not observe a statistically significant association between chronic HBV infection and severe COVID-19 outcomes (15). Notably, most previous studies have primarily focused on chronic HBV infection or overall HBV infection, whereas evidence regarding resolved HBV infection remains limited. These inconsistent findings suggest that the impact of HBV infection on COVID-19 outcomes may vary according to HBV infection status, viral replication activity, liver disease stage, cirrhosis, antiviral treatment, and the specific clinical outcomes assessed. Our study suggested that COVID-19 patients with current HBV infection did not have an increased incidence of liver injury, consistent with previous studies (15, 18, 21). Although one COVID-19 patient with current HBV infection died, no liver injury was found in this patient, and a lethal cytokine storm might be the main cause of the death.

Compared with previous studies, our study focused specifically on resolved HBV infection rather than on chronic or overall HBV infection. This distinction is clinically relevant because resolved HBV infection is generally defined by HBsAg-negative and anti-HBc-positive, reflecting previous HBV exposure rather than active HBV infection. Most previous studies have mainly evaluated clinical outcomes, including mortality, severe COVID-19, ICU admission, and liver injury, while evidence regarding inflammatory cytokine profiles in patients with resolved HBV infection remains limited. Our findings therefore extend previous observations by suggesting that resolved HBV infection might be associated with an enhanced systemic inflammatory response, as indicated by higher IL-6, sIL-2R, and TNF-*α* levels.

Abnormal liver function was often detected in COVID-19 patients (22). In the present study, liver injury was more frequent in COVID-19 patients with resolved HBV infection compared to those with non-HBV infection. Systemic inflammatory response could be a fuel for liver injury in patients with COVID-19 (23). However, patients with high levels of inflammatory cytokines did not always develop liver injury in our study. Among the eight deceased COVID-19 patients with resolved HBV infection, elevated levels of proinflammatory cytokines, including IL-6, sIL-2R, and TNF-*α*, were observed, whereas liver injury occurred in only three patients. Although liver injury may contribute to systemic inflammation, these findings suggest that it may not fully explain the enhanced cytokine responses observed in COVID-19 patients with resolved HBV infection, indicating that additional immunological mechanisms may also play a role.

Compared with patients with non-HBV infection, the levels of proinflammatory cytokines, such as IL-6, IL-8, TNF-α, and sIL-2R, were substantially increased in patients with resolved HBV infection. Patients with resolved HBV infection were more likely to receive glucocorticoid treatment, which also indicates the excessive inflammatory responses in COVID-19 patients with resolved HBV infection. Cytokine storm is one of the most important immunopathological processes that is responsible for the progression of COVID-19 (24).

In the present study, the mortality of COVID-19 patients with resolved HBV infection was higher than that of patients with non-HBV infection. Most deceased cases of COVID-19 with resolved HBV infection were found to have excessive levels of proinflammatory cytokines, except for one who did not have cytokine tests in time. Moreover, all eight deceased cases with resolved HBV infection were elderly patients with ages over 60, and 7 of them had underlying comorbidities like diabetes, hypertension, and coronary heart disease. Therefore, resolved HBV infection, together with old age and comorbidities, may be risk factors for COVID-19 patients.

A possible reason for the excessive inflammatory responses in COVID-19 patients with resolved HBV infection might be the potential immune system disorders left after spontaneous elimination of HBV (6). Although resolved HBV infection indicates apparent viral control rather than active HBV replication, anti-HBc positivity reflects prior HBV exposure and may be associated with persistent immune alterations or immune memory (4). Recent bioinformatics studies suggested that HBV infection and COVID-19 may share immune-related pathways, including T-cell activation and interferon-*γ*-related immune regulation (25). Although these findings do not directly establish the mechanism underlying the resolution of HBV infection and COVID-19, they support the possibility that prior HBV exposure may influence host inflammatory responses during SARS-CoV-2 infection. In this regard, COVID-19 patients with resolved HBV infection might have an altered immune response compared to patients with non-HBV infection. The underlying mechanism needs further investigation. Considering the large population of resolved HBV infection (3) and the continuous global burden of COVID-19, HBV serological tests are encouraged for patients with COVID-19. Moreover, patients with resolved HBV infection should receive greater attention after diagnosis.

The limitation of the current study is that it was merely a single-center, retrospective study with a small cohort. Thus, a large sample multicenter study is warranted for further investigation.

## Conclusion

In conclusion, our findings suggest that COVID-19 patients with resolved HBV infection may exhibit enhanced systemic inflammatory responses compared with patients without evidence of HBV infection. These observations highlight the potential clinical relevance of HBV serological status in assessing COVID-19 patients, particularly in individuals with current or resolved HBV infection. Furthermore, elderly patients with resolved HBV infection and multiple comorbidities appeared to be at increased risk of mortality, although further studies are required to validate these findings.

## Data Availability

The original contributions presented in the study are included in the article/Supplementary material, further inquiries can be directed to the corresponding authors.
