# Supplementary material for: Resolved HBV infection is an independent risk factor for enhanced systemic inflammatory responses in COVID-19 patients
Source: Front Med (Lausanne). 2026 Jul 28;13:1835022. doi: 10.3389/fmed.2026.1835022 (PMC13459760; doi:10.3389/fmed.2026.1835022)
Supplement: Supplementary file 1 [file Supplementary_file_1.docx]

**Resolved HBV infection is an independent risk factor for excessive inflammatory responses in COVID-19 patients**

Yifan Xu^1,2 †^, Jia Lu^1,2 †^, Wei Chen^3 †^, Xiaoxiang Yan^4 *^, Weiguo Hu^5 *^, Xiaogang Xiang^1,2 *^

Yifan Xu, yifanxu19991007@163.com

Jia Lu, 1931954598@qq.com

Wei Chen, monbox007@hotmail.com

Xiaoxiang Yan, cardexyanxx@hotmail.com

Weiguo Hu, wghu@rjh.com.cn

Xiaogang Xiang, shine-xxg@163.com

^1^Department of Infectious Diseases, Ruijin Hospital, Shanghai Jiao Tong University School of Medicine, China;

^2^Translational Laboratory of Liver Diseases, Department of Infectious Diseases, Ruijin Hospital, Shanghai Jiao Tong University School of Medicine, China;

^3^Department of Pulmonary and Critical Care Medicine, Ruijin Hospital, Shanghai Jiao Tong University School of Medicine, China;

^4^Department of Cardiology, Ruijin Hospital, Shanghai Jiao Tong University School of Medicine, China;

^5^Medical Center on Aging of Ruijin Hospital, Department of Geriatrics, Ruijin Hospital, Shanghai Jiao Tong University School of Medicine, China;

†These authors have contributed equally to this work

*** Correspondence:**

Xiaogang Xiang, shine-xxg@163.com

Weiguo Hu, wghu@rjh.com.cn

Xiaoxiang Yan, cardexyanxx@hotmail.com

**Supplementary material**

**Supplementary Figure S1**

**Supplementary Figure 1. Flow diagram for the inclusion of COVID-19 patients.** There were 314 patients with COVID-19 admitted to Tongji hospital from January 20, 2020 to March 30, 2020, while 99 patients were excluded for unavailable results of HBV tests. 215 patients were finally included in the present study. Fifteen patients with COVID-19 had current HBV infection, 134 patients had resolved HBV infection and 66 had non-HBV infection.

**Supplementary Figure S2**

**Supplementary Figure 2. The relationships between relative quantitative anti-HBc and the levels of IL-6, sIL-2R, TNF-α and IL-8.** The levels of IL-6, TNF-α and sIL-2R were positively correlated with relative quantitative anti-HBc.

**Supplementary Table S1.** **Clinical characteristics of 10 deceased COVID-19 patients in the present cohort.**

|  | **Patient 1** | **Patient 2** | **Patient 3** | **Patient 4** | **Patient 5** | **Patient 6** | **Patient 7** | **Patient 8** | **Patient 9** | **Patient 10** |
| --- | --- | --- | --- | --- | --- | --- | --- | --- | --- | --- |
| **HBV status** | Current HBV infection | Resolved HBV infection | Resolved HBV infection | Resolved HBV infection | Resolved HBV infection | Resolved HBV infection | Resolved HBV infection | Resolved HBV infection | Resolved HBV infection | Non-HBV infection |
| **Age** | 69 | 74 | 72 | 66 | 70 | 68 | 66 | 76 | 61 | 64 |
| **Gender** | Male | Male | Male | Male | Female | Male | Female | Female | Male | Male |
| **Diagnostic classification** | Critical | Critical | Critical | Critical | Critical | Critical | Critical | Critical | Critical | Critical |
| **Anti-HBc (S/CO)** | 10.51 | 7.75 | 2.03 | 5.65 | 9.33 | 1.07 | 4.67 | 9.31 | 3.15 | 0.87 |
| **Peak ALT (U/L)** | 10 | 107 | 44 | 78 | 25 | 31 | 24 | 738 | 40 | 432 |
| **Peak AST (U/L)** | 60 | 211 | 43 | 48 | 40 | 34 | 36 | 4171 | 125 | 289 |
| **Total bilirubin (μmol/L)** | 6.1 | 16.7 | 30.8 | 10.6 | 16.9 | 6.2 | 7.7 | 67.8 | 46.1 | 27.8 |
| **Peak CRP (mg/L)** | 270.1 | 274.1 | 181.9 | 164.5 | 62.4 | 108.2 | 320 | 150.4 | 168.7 | 242.9 |
| **Peak ferritin (mg/dL)** | 1235.8 | 2578.7 | 971 | / | / | 785.5 | 4946.9 | 27589 | 5244.4 | 6543.7 |
| **IL-6 (pg/mL)** | 1224 | 162.9 | 460.4 | / | 24.26 | 243.5 | 5000 | 2449 | 3525 | 1166 |
| **IL-8 (pg/mL)** | 147 | 67.2 | 34.9 | / | 20.9 | 1283 | 3059 | 852 | 129 | 42.3 |
| **sIL-2R (U/mL)** | 6491 | 1301 | 299 | / | 1258 | 2314 | 3416 | 4925 | 1087 | 2245 |
| **TNF-α (pg/mL)** | 36.8 | 9.9 | 23.7 | / | 13.7 | 40.6 | 223 | 55.8 | 41.7 | 16.7 |
| **Comorbidity** | Hypertension, Tuberculosis | Diabetes | Hypertension, Parkinson | Hypertension | Hypertension, COPD | Hepatitis C | Diabetes, Hypertension, Coronary heart disease, Hepatitis C | Hypertension, Coronary heart disease | / | / |

**Note:** Liver function, inflammatory cytokines, and clinical characteristics of the 10 deceased COVID-19 patients in the present cohort are shown. / indicates data not available.

**Supplementary Table S2. Factors associated with IL-8 levels in patients.**

|  | **Univariate Analysis** | |
| --- | --- | --- |
| Parameters | OR (95% CI) | *p* value |
| Non-HBV | Ref |  |
| Resolved HBV infection | 2.079 (0.658-6.568) | 0.2123 |
| Age | 1.025 (0.986-1.065) | 0.2088 |
| Male sex | 0.928 (0.354-2.431) | 0.8784 |
| Diabetes | 0.965 (0.300-3.097) | 0.9519 |
| Hypertension | 0.726 (0.262-2.013) | 0.5387 |
| Coronary heart disease | 1.448 (0.384-5.460) | 0.5847 |
| Stroke | 0.000 (0.000-NA) | 0.9996 |
| COPD | 0.914 (0.109-7.635) | 0.9335 |
| Anti-virus drug | 0.779 (0.208-2.913) | 0.7100 |
| Anti-bacterial drug | 3.798 (1.207-11.952) | **0.0225** |

**Note:** Logistic regression was performed on factors associated with elevated IL-8 levels. Only univariable results are shown because resolved HBV infection did not meet the prespecified criterion for entry into the multivariable model. *p* < 0.05 was considered statistically significant. Bold values indicate statistically significant *p* values (*p* < 0.05).
